# Sickness absence among young employees in private and public sectors with a history of depression and anxiety

**DOI:** 10.1038/s41598-022-21892-z

**Published:** 2022-11-04

**Authors:** Jurgita Narusyte, Annina Ropponen, Mo Wang, Pia Svedberg

**Affiliations:** 1 Division of Insurance Medicine, Department of Clinical Neuroscience, Karolinska Institutet, 171 77 Stockholm, Sweden; 2 Finnish Institute of Occupational Health, Helsinki, Finland

**Keywords:** Risk factors, Psychiatric disorders

## Abstract

The aim was to investigate occurrence and duration of sickness absence (SA) among young employees with previous depression/anxiety in private and public sectors. This population-based prospective study included 11,519 Swedish twin individuals of age 19–29 years that were followed regarding SA during 2006–2016. Data on previous depression/anxiety came from two screening surveys in 2005. Data on SA and employment sector were received from national registries. Descriptive statistics and logistic regression were used, also controlling for familial factors. Proportion of employees with SA was significantly higher among those with, as compared to those without, previous depression/anxiety, regardless the employment sector. Individuals with previous depression/anxiety had increased risk for future SA, in both private (OR 2.25, 95% CI 1.90–2.66) and public sectors (OR 2.10, 95% CI 1.73–2.54). Familial factors played a role in the association among employees in the private sector. A higher proportion of long-term SA was observed among employees with previous depression/anxiety in the private as compared to the public sector. To conclude, previous depression/anxiety tends to increase risk for SA among young employees in both employment sectors, whereas long-term SA seemed to be more prevalent among those in the private as compared to the public sector.

## Introduction

Different rates of sickness absence (SA) among those employed in private and public sectors have been previously reported in several countries^1–3^. However, knowledge is still scarce on rates of SA among young employees in private and public sectors, a group of individuals where SA due to mental disorders is most common both in Sweden and other Nordic countries^4–9^. Understanding factors contributing to SA differences between young adults employed in private and public sectors may help to identify factors affecting future SA as well as promoting working force participation.

Mental ill-health among young people is a great concern in the society today as prevalence continues to increase among those at 16–29 years of age in Sweden^10^ and rates are also high in other countries^11,12^. Most mental disorders start before age of 24 years^13^, indicating adolescence and early adulthood as developmental periods of increased liability for onset of mental disorders. Several mental disorders with onset in adolescence, including depression and anxiety, are denoted by continuity into adulthood^14–16^, which suggest that mental disorders may last for a long period and affect functioning in adulthood. Previous studies report on increased risk for disadvantageous outcomes in adulthood including health, economy, and labor market participation^17–20^ among those with a history of depression and/or anxiety in adolescence. Findings from a few studies on mental disorders in adolescence and future SA suggest that depressive and anxiety symptoms in early life years tend to increase the risk for SA later in adulthood^21,22^. However, whether the association is valid for employees both in private and public sectors is still unknown.

According to previous research, factors contributing to higher rates of SA among those employed in the public sector as compared to the private sector include sex, age, health status or size of the company^2,23^. Less is known, though, on whether SA rates are comparable between private and public sectors when studied in specific age groups, for example, young or older employees. A recent study reported that young adults employed in public sector had an increased risk for future SA due to common mental disorders as compared to those in private sector^7^. Having in mind the early start and continuity of mental disorders, a question is whether mental ill-health in early years may explain the association. Knowledge on SA occurrence, duration, and diagnoses among young employees with previous history of mental ill-health is lacking in both employment sectors, despite its importance for employers and occupational or other health care specialists.

Using data on twins provides a unique opportunity to account for confounding due to unmeasured early life factors including genetics and environment shared in a family. Genetic factors tend to explain approximately 30–40% of the variance in depression and anxiety as well as for SA^24–27^. This suggests that associations between depression/anxiety and SA may be partly confounded by shared genetics, which, if not controlled for, may undermine the importance of environmental influences.

The aim of the study was, by combining survey and register data, to investigate occurrence of the first SA including diagnoses and duration among young adults employed in private and public sectors, depending on their previous history of depression/anxiety in emerging adulthood.

## Material and methods

### Participants

The prospective longitudinal population-based study included all the twins in the Swedish Twin project Of Disability pension and Sickness absence (STODS) that were born 1975–1986 in Sweden and that were identified through the Swedish Twin Registry (STR). Depending on the birth year, the twins were invited by STR to participate in two different surveys.

Twins born in 1985–1986 participated in a study called Twin study of Child and Adolescent Development (TCHAD), which is a longitudinal study of development of physical and mental health from childhood to adulthood^28^. The twins and their parents were contacted at four times in TCHAD. In the present study, twins’ responses in 2005 were used, that is, when twins were 19–20 years old.

Twins born in 1959–1985 participated in a comprehensive survey The Study of Twin Adults: Genes and Environment (STAGE) in 2005^29^. In the present study, we included those twins who were born 1975–1985 and were 20–30 years old in 2005.

All twins who participated in TCHAD or STAGE were followed from year 2006 until year 2016 regarding occurrence of SA. Excluded were twins that were granted disability pension as they were no longer at risk for SA, emigrated, or died during the follow up.

The final sample included 11 519 twins, of which 1743 twins participated in TCHAD and 9776 twins participated in STAGE. Of these, there were 5206 monozygotic (MZ) twins, 3369 same-sex dizygotic (DZ) twins, and 3564 opposite-sex DZ twins.

### Ethical approval

The study was approved by Regional Ethical Review Board of Stockholm (DNR 2010/1346-32/5, 2010-09-23). All methods were performed in accordance with the relevant guidelines and regulations. Informed consent was obtained from all participants in TCHAD (or their parents and/or legal guardians) and STAGE surveys. The subjects in STODS have previously been informed of registry-based linkages.

### Measures

#### Depression and anxiety

In TCHAD, depression and anxiety were assessed by Internalizing problems scale in Young Adult Self-Report (YASR), which is a reliable and valid instrument for assessment of functioning and mental health problems in adults^30^. In the Internalizing problems scale, the syndromes are grouped as Anxious/Depressed (18 items), Withdrawn (9 items), and Somatic complaints (12 items). In the current study, presence of depression/anxiety was denoted by clinical scores over 65 in Internalizing problems scale. In STAGE, the measure of depressive and of generalized anxiety disorders was based on the Structured Clinical Interview for DSM-IV Disorders (SCID)^31^. Criteria A, C, and E had to be fulfilled for the participant to be classified as having a positive history of MD. The individuals were considered as positive for a history of GAD if they reported on excess worry or anxiety that had lasted for at least 6 months (DSM-IV criterion A) and at least three of five symptoms (except “difficulty concentrating or mind going blank”) that were associated with worry and anxiety and had lasted for at least six months (criterion C). Finally, a common dichotomous variable was created to denote presence of depression/anxiety (yes/no) in TCHAD or STAGE, which was used in further analyses.

### Sickness absence

Data on SA (date, duration, and diagnoses) were obtained from the National Social Insurance Agency MiDAS database. A dichotomous variable was created for the occurrence of the first incident SA spell (yes/no) during follow up. Duration of the first SA spell during the follow up was stratified in three categories: 1–30 days, 31–90 days, and more than 90 days. SA diagnoses followed ICD-10 classification on chapter level.

### Occupational sector

Occupational sector was identified in the LISA-database from Statistics Sweden. Private sector included individuals employed at private companies, whereas public sector covered individuals employed by state and municipality. Individuals were classified as employed at public or private sector after being employed in either of sectors for at least three years in total during the follow up. If an individual worked in both sectors for an equal number of years, an employment sector during the last years of the follow-up was chosen.

### Statistical analyses

Descriptive statistics including frequencies and proportions were calculated for the whole sample and separately for each occupational sector, history of depression/anxiety, and sex. The difference between groups were tested by Chi^2^ test. The five most common SA diagnoses SA were depicted on diagnose ICD 10-chapter level. Odds ratios (OR) with 95% Confidence Intervals (CI) for SA during the follow-up among those with a previous history of depression/anxiety were calculated by logistic regression adjusting for intrapair twin covariation. The analyses were also adjusted for age, sex, and education level at baseline. Conditional logistic regression analyses were run in a subsample of discordant twin pairs to examine the influence of familial factors on the associations. Discordant twin pairs included the same-sex twin pairs, where one twin in a pair had a SA spell during the follow-up, whereas the co-twin did not. Familial factors are suggested to be of importance if ORs computed in a subsample of discordant twins are different from the ORs calculated in the whole sample. All statistical analyses were performed using SAS software version 9.4^32^.

## Results

Descriptive statistics of the study sample are presented in Table 1. Most young employees (mean age 24 years) in the public sector were women (73%), whereas 44% of those employed in the private sector were women. A higher proportion of those working in the public sector had 12 years or longer education (73%) than the private sector (51%). The prevalence of depression/anxiety was higher among those employed in the public sector (17%) as compared to the private sector (12%). Higher proportion of those employed in public sector (53%) had at least one incident SA spell during the follow-up as compared to those in private sector (42%). The duration of the first SA spell during the follow-up differed significantly between these two sectors. In total, 5635 individuals (44%) had at least one SA spell during the follow-up period, of these 3868 (55%) were women and 1767 (31%) men (data not shown).

**Table 1:** Descriptive statistics of the study sample by occupational sector.

	Public (n = 4113)		Private (n = 7406)		*p*-value*
	N	%	n	%	
**Women**	3019	73	3280	44	< 0.0001
**Mean age (sd) at baseline 2005**	24 (4)		24 (4)		
**Education**					< 0.0001
- < 12 years	1110	27	3589	48	
- > = 12 years	3001	73	3813	51	
**Depression/Anxiety**	545	17	667	12	< 0.0001
**First incident SA spell during 2006–2016**	2190	53	3133	42	< 0.0001
**Duration of the first SA during 2006–2016, in days**					< 0.0001
1–30	1416	65	1883	61	
31–90	500	23	742	24	
> 90	252	12	483	16	

*p-value for the difference between public and private sectors.

The occurrence of the first SA was higher among those employed in the public as compared to the private sector despite the previous history of depression/anxiety (Table 2). Additional analyses showed that the occurrence of SA was higher among those young employees with previous history of depression/anxiety, in both public and private sectors (*p* < 0.01, not in Table 2). However, SA duration was not significantly different between the sectors regardless of previous history of depression/anxiety. Further, SA duration differed significantly between those with and without previous depression/anxiety in each of the sectors (*p* < 0.01, not in Table 2). In both public and private sectors, higher proportion of SA spells longer than 90 days was observed among those with previous history of depression/anxiety (16% vs. 11% in public and 23% vs. 13% in private sector).

**Table 2:** Occurrence and duration of SA in public and private occupational sectors among those with and without previous history of depression/anxiety.

	Previous depression/anxiety					No previous depression/anxiety				
	Public (n = 545)		Private (n = 667)		*p*-value*	Public (n = 2669)		Private (n = 4687)		*p*-value*
	n	%	n	%		N	%	n	%	
**First incident SA during 2006–2016**	373	68	400	60	0.002	1338	50	1844	39	< 0.0001
**Duration of the first SA during 2006–2016, in days**					0.05					0.09
1–30	216	59	211	53		880	67	1155	63	
31–90	94	25	96	24		298	23	433	24	
> 90	59	16	92	23		144	11	239	13	

*p-value for the difference between public and private sectors.

Logistic regression analysis showed that those young employees with previous depression/anxiety had a significantly increased risk for SA during the follow-up in both private (OR 2.25, 95% CI 1.90–2.66) and public sector (OR 2.10, 95% CI 1.73–2.54) as compared to those without previous depression/anxiety (Table 3). The associations retained the direction but decreased in magnitude after adjusting for age, sex, and education. After controlling for familial factors in the subsample of discordant twins, the association did not reach statistical significance among those employed in the private sector (OR 1.37; 95% CI 0.96–1.95). The estimate of the association remained statistically significant when controlling for familial confounding among those employed in the public sector (OR 1.93, 95% CI 1.24–3.00).

**Table 3:** Odds ratios (OR) for occurrence of the first spell of sickness absence during 2006 2016 with previous history of depression/anxiety among those employed in private and public sectors.

	Private (n = 5354)		
	Crude (n = 5354)	Adjusted (n = 5354)	Discordant twins (n = 1046)
	OR (95% CI)	OR (95% CI)	OR (95% CI)
No previous depression/anxiety (ref)	1	1	
Previous depression/anxiety	2.25 (1.90–2.66)	1.98 (1.66–2.36)	1.37 (0.96–1.95)

	Public (n = 3214)		
	Crude (n = 3214)	Adjusted (n = 3214)	Discordant twins (n = 655)
No previous depression/anxiety (ref)	1	1	1
Previous depression/anxiety	2.10 (1.73–2.54)	1.91 (1.56–2.34)	1.93 (1.24–3.00)

*Adjusted for age, sex, and education.

**Figure 1:**
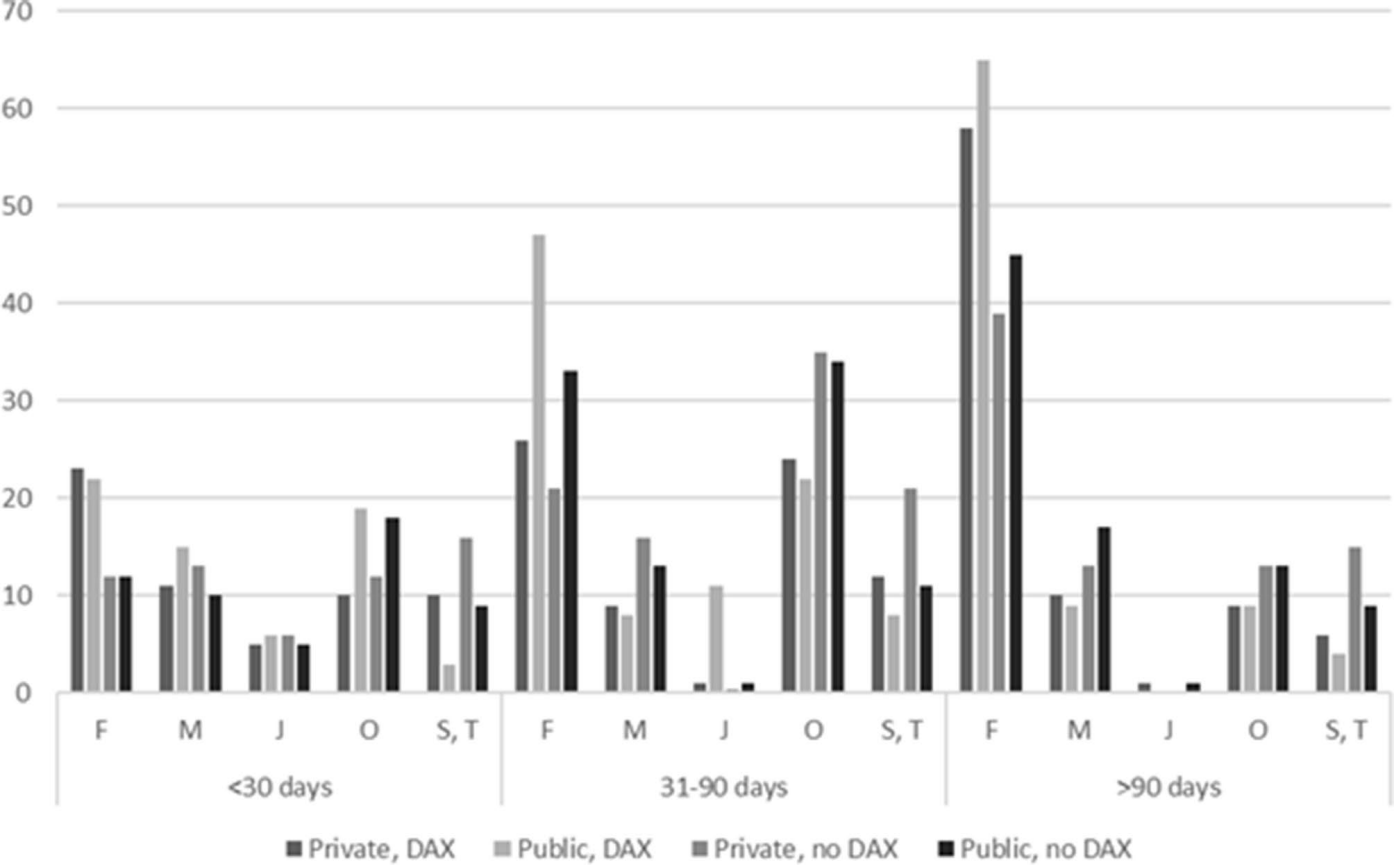
Most common diagnoses for the first SA spell of different durations among employees in private and public sector, with/without previous history of depression/anxiety (DAX). ICD-10 chapter F: mental disorders (F00–F99), chapter M: musculoskeletal disorders (M00–M99), chapter J: diagnoses of the respiratory system (J00–J99), chapter O diagnoses related to pregnancy and delivery (O00–O99), and chapters S and T: diagnoses related to injuries (S00–T98).

The five most common diagnoses for SA spells of different duration are presented in Fig. 1. The largest group of diagnoses for SA spells > 90 days were related to mental disorders (ICD-10, chapter F), regardless of occupational sector and previous history of depression/anxiety. However, the proportion of SA spells > 90 days due to mental disorders were higher among those with previous history of depression/anxiety as compared to those without such a history. The proportion of SA spells due to mental disorders between 31 and 90 days was comparable to the proportion of SA due to pregnancy and delivery related diagnoses (chapter O, ICD-10). Overall, the proportion of SA spells due to mental disorders tended to be higher among those employed in the public as compared to the private sector except SA spells ≤ 30 days, where similar proportions of SA due to mental disorders were observed in both private and public sectors. The proportion of SA due to diagnoses related to injuries (chapters S and T, ICD-10) was consequently higher among those employed in private sector, regardless the duration of SA and previous history of depression/anxiety.

Additional analyses were performed to study SA occurrence among women and men. In both private and public sectors, the occurrence of SA was significantly higher among both women and men with previous history of depression/anxiety as compared to those without (Table S1). Among those who have had at least one SA spell during follow-up, SA spells ≤ 30 days were more common among those with no previous depression/anxiety, in both sectors and both sexes. A higher proportion of both women and men employed in the private sector had SA spells over 90 days compared to those employed in the public sector (Table S1).

## Discussion

In this population-based prospective study of young employees in public and private sectors we found that SA was higher among those with, as compared to those without, previous history of depression/anxiety. Those with previous depression/anxiety experienced approximately the same risk for future SA in both private and public sector. However, among those with previous depression/anxiety, a higher proportion of young employees had long-term SA spells in the private as compared to the public sector. This finding adds an important aspect when discussing SA rates in public as compared to private sectors and especially among young employees^1,23^.

Young employees with a history of depression/anxiety were shown to have approximately the same risk for future SA in both private and public sectors. However, several previous studies reported higher SA rates in public than private sector in different age groups, with one explanation being that the difference in SA rates may be attributable to employee’s health^1,2,7,23^. Our findings of young employees suggest that depression/anxiety in early years may explain the different SA rates in public and private sectors. The inflated risk for SA among those with, as compared to those without, previous depression/anxiety is also in line with previous findings on continuity of mental disorders^14,16^ and increases awareness of long-term liability for work incapacity due to mental disorders with initiation in adolescence and emerging adulthood.

Our results regarding SA duration are partly in line with findings from previous studies (3, 7). Higher prevalence of SA spells ≤ 30 days was observed among young employees in the public sector as compared to the private, similarly to a previous report^2^. The prevalence of long-term SA (> 90 days) in the current study was higher among those employed in the private sector, primarily among those with previous depression/anxiety. This differs from findings reported earlier, where employees in the public sector were shown to have increased risk for long-term SA due to common mental disorders among young employees^7^ or no difference between the sectors was observed at all in a sample of middle-aged employees^2^. Inconsistency in findings regarding long-term SA may be attributable to different features of study samples. For example, in this study, SA duration was examined separately for study participants with and without previous history of depression/anxiety whereas in earlier studies that has rarely been done^2,7^. Our finding also points to the direction that young adults with previous mental ill-health who are working in private sector seem to be a vulnerable group for long-term SA.

The occurrence of short-term SA (≤ 30 days) was higher among those young employees with no previous depression/anxiety, whereas the prevalence of long-term SA (> 90 days) was higher among those with previous depression/anxiety, a result valid in both sexes and both employment sectors. According to previous studies, SA tends to be of short duration in younger ages and increase with increasing age^33–35^. Our results suggest that this may be valid for those young employees with no previous history of depression or anxiety. We could also show that the highest proportion of long-term SA diagnoses were related to mental disorders, and the prevalence was higher among those with previous depression/anxiety as compared to those without. This is in line with previous reports that, for example, depressive symptoms in adolescence tend to be followed by recurrent episodes in adulthood^36^, also increasing risk for SA including long-term SA^1,33,37,38^.

The association between previous history of depression/anxiety and SA among young employees in the private sector, but not the public, seem to be partly explained by familial factors. The different finding between the employment sectors suggests that SA diagnoses among young employees in private and public sectors tend to differ. That is, those employed in the private sector seem to be more likely to be on SA due to mental diagnoses than those in the public sector, where a broader panorama of SA diagnoses exists. This is partly in line with findings from a previous study, where genetic factors contributing to disability pension due to mood and neurotic disorders were to a large part not explained by genetic influences on depression/anxiety^39^. Similarly, genetic variance in SA due to mental disorders was not completely explained by previous anxiety and mood disorders in young adults in another study^40^.

### Strengths and limitations

Strengths of the study include extensive survey data in combination with national register data i.e., no loss to follow-up and no reporting bias for the study outcomes (SA). Furthermore, until now, such population-based studies of young employees have been lacking although long-term consequences of mental ill-health have been of interest^19^. Limitations include different evaluation of depression in two survey studies used, TCHAD and STAGE. Depression and anxiety as evaluated in TCHAD may include less severe symptoms than diagnoses of major depression and generalized anxiety disorders in STAGE. However, we used clinical scores in TCHAD to identify individuals with depressive/anxiety symptoms in a clinical range meaning that the severity of the symptoms should be comparable to DSM-IV diagnostic criteria. Assessment of depression/anxiety was based on self-report data which may be a source for recall bias. Also, a potential limitation may be related to misclassification of the employment sector since the sector categorization was based on the total number of years of work in each of the sectors during the study follow-up. Further, since only SA spells longer than 14 days are reported in national registries in Sweden, we had no possibility to study the occurrence of short SA spells (≤ 14 days). Another limitation is related to sample size. Despite the large study sample size, the number of individuals in different SA duration categories as well as number of complete pairs in the discordant twin analyses, was quite small. Thus, caution needs to be taken when interpreting results that need to be replicated in future studies. However, we expect these results to be generalizable at least in the Nordic countries with similar welfare system and employment among young people^41^.

## Conclusions

Previous history of depression/anxiety is associated with future SA among young employees regardless of the employment sector, whereas long-term SA seemed to be more prevalent among employees in the private sector as compared to the public sector. The results indicate a need for awareness for work incapacity of young employees with previous mental ill-health, both in the private and public sectors.

## Supplementary Information

Supplementary Information.

## Data Availability

Restrictions apply to the availability of the data used in this study based on the Swedish Twin project of Disability pension and Sickness absence (STODS), which were used with ethical permission for the current study and therefore are not publicly available. Legal restrictions set out in the General Data Protection Regulation, the Swedish law SFS 2018:218, the Swedish Data Protection Act, the Swedish Ethical Review Act, and the Public Access to Information and Secrecy Act are applied. The data that support the findings of this study are available from the original sources: the Swedish Twin Registry, Statistics Sweden, the Swedish Social Insurance Agency and the Swedish National Board of Health and Welfare.
