# Supplementary material for: Sickness absence among young employees in private and public sectors with a history of depression and anxiety
Source: Sci Rep. 2022 Nov 4;12:18695. doi: 10.1038/s41598-022-21892-z (PMC9636248; doi:10.1038/s41598-022-21892-z)
Supplement: Supplementary file 1 — Supplementary Information. [file 41598_2022_21892_MOESM1_ESM.pdf]

Table S1. **Occurrence and duration of SA in men and women with and without previous history of depression/anxiety in public and private occupational sectors.**

	**Men**									
	**Private**					**Public**				
	Previous depression/anxiety (n=228)		No previous depression/anxiety (n=2655)		**p-value**	Previous depression/anxiety (n=78)		No previous depression/anxiety (n=739)		**p-value**
	n	%	n	%		N	%	n	%	
**At least one SA**	111	49	799	30	<0.001	36	54	220	30	0.003
**First SA duration,** days					<0.001					0.02
1-30	56	50	491	62		18	50	146	67	
31-90	22	20	185	23		9	25	51	23	
>90	33	30	114	14		9	25	21	10	
	**Women**									
	(n=439)		(n=2032)		**p-value**	(n=467)		(n=1930)		**p-value**
	n	%	n	%		n	%	n	%	
**At least one SA**	289	66	1045	51	<0.001	337	72	1118	58	<0.0001
**SA duration,** days					<0.001					0.04
1-30	155	54	664	64		198	59	734	66	
31-90	74	26	248	24		85	26	247	22	
>90	59	20	125	12		50	15	123	11	

* p-value for the difference between those with and without previous depression/anxiety
